# Hermansky-Pudlak syndrome type 2 manifests with fibrosing lung disease early in childhood

**DOI:** 10.1186/s13023-018-0780-z

**Published:** 2018-03-27

**Authors:** Meike Hengst, Lutz Naehrlich, Poornima Mahavadi, Joerg Grosse-Onnebrink, Suzanne Terheggen-Lagro, Lars Høsøien Skanke, Luise A. Schuch, Frank Brasch, Andreas Guenther, Simone Reu, Julia Ley-Zaporozhan, Matthias Griese

**Affiliations:** 1 Ludwig-Maximilians University, Dr von Haunersches Kinderspital, German Center for Lung Research (DZL), Lindwurmstr. 4, 80337 Munich, Germany; 2 University Hospital Gießen and Marburg, German Center for Lung Research, Feulgenstr. 12, 35385 Gießen, Germany; 3 Department of Internal Medicine, Justus-Liebig University, German Center for Lung Research, Klinikstrasse 36, 35392 Giessen, Germany; 4 University Hospital Münster, Albert-Schweitzer-Campus 1, 48149 Münster, Germany; 5 Erasmuc MC, University Medical Center Rotterdam, S’Gravendijkwal 230, 3015 Rotterdam, The Netherlands; 6 St.Olav’s University Hospital, Trondheim, Norway; 7 Klinikum Bielefeld Mitte, Institut für Pathologie, Teutoburger Straße 50, 33604 Bielefeld, Germany; 8 Member of the European IPF Network, Lung Clinic Waldhof-Elgershausen, Greifenstein, Germany; 9 Ludwig-Maximilians University, Institute of Pathology, Thalkirchnerstr. 36, 80337 Munich, Germany; 10 Department of Radiology, Ludwig-Maximilians University, Lindwurmstr. 4, 80337 Munich, Germany

**Keywords:** Hermansky-Pudlak syndrome type 2, Childhood, Pulmonary fibrosis, Tachydyspnea, Pulmonary phenotype

## Abstract

**Background:**

Hermansky-Pudlak syndrome (HPS), a hereditary multisystem disorder with oculocutaneous albinism, may be caused by mutations in one of at least 10 separate genes. The HPS-2 subtype is distinguished by the presence of neutropenia and knowledge of its pulmonary phenotype in children is scarce.

**Methods:**

Six children with genetically proven HPS-2 presented to the chILD-EU register between 2009 and 2017; the data were collected systematically and imaging studies were scored blinded.

**Results:**

Pulmonary symptoms including dyspnea, coughing, need for oxygen, and clubbing started 3.3 years before the diagnosis was made at the mean age of 8.83 years (range 2-15). All children had recurrent pulmonary infections, 3 had a spontaneous pneumothorax, and 4 developed scoliosis. The frequency of pulmonary complaints increased over time. The leading radiographic pattern was ground-glass opacities with a rapid increase in reticular pattern and traction bronchiectasis between initial and follow-up Computer tomography (CT) in all subjects. Honeycombing and cysts were newly detectable in 3 patients. Half of the patients received a lung biopsy for diagnosis; histological patterns were cellular non-specific interstitial pneumonia, usual interstitial pneumonia-like, and desquamative interstitial pneumonia.

**Conclusions:**

HPS-2 is characterized by a rapidly fibrosing lung disease during early childhood. Effective treatments are required.

**Electronic supplementary material:**

The online version of this article (10.1186/s13023-018-0780-z) contains supplementary material, which is available to authorized users.

## Background

Hermansky Pudlak syndrome is a rare hereditary multisystem disorder first described in 1959. More than 50% of all worldwide cases are identified in individuals from Puerto Rico where HPS has an estimated frequency of 1:1800 [1, 2]. Clinically the syndrome is characterized by oculocutaneous albinism, a bleeding diathesis due to platelet storage deficiency [1, 2], and other manifestations which may include neutropenia, a granulomatous colitis, or pulmonary fibrosis.

Genotypic analysis over the last decade allowed differentiating at least 10 separate forms of HPS, due to mutations in different genes [3]. All entities have in common defects in intracellular protein trafficking and the biogenesis of lysosome-related organelles like melanosomes or platelet dense granules [2].

Pulmonary fibrosis has not been described in HPS-3 and HPS-5 through HPS-10, which are all very rare. HPS-1 is the most common subtype and characteristically develops a severe and progressive pulmonary fibrosis in almost all cases. Usually middle-aged adults and rarely late adolescents are affected by fibrosis; however, so far, children are not described [2–4]. HPS-4 has been documented in less than 10 patients, few of which had pulmonary fibrosis [5, 6].

The HPS-2 subtype is also very rare, with less than 40 cases reported worldwide [4, 7–10]. HPS-2 is caused by mutations in the *AP3B1* gene, inherited in an autosomal recessive way and distinguished from the other forms of HPS by the presence of neutropenia that can lead to severe respiratory infections and that is responsive to granulocyte colony-stimulating factor [1]. Among the few patients described, development of an interstitial lung disease (ILD) has been mentioned in 30 to 50% [1, 7]; details on the pulmonary phenotype have been described in four cases [4]. Potential mechanisms causing pulmonary disease in HPS-2 are poorly understood. It has been suggested that altered *AP3B1* gene product within alveolar epithelial type II cells leads to defective intracellular processing of surfactant proteins B and C (SP-B, SP-C), endoplasmic reticulum-stress, apoptosis, and a fibrotic lung phenotype [1].

The aim of this study was to describe the pulmonary phenotype of HPS-2 in children and to further investigate the presence and the possible role of cellular stress and apoptosis in patient-derived material.

## Methods

### Patients, diagnosis, and follow up

Patients were recruited from the chILD-EU register and biobank and the kids’ lung register collecting diffuse parenchymal lung diseases [11, 12]. Among the children included between 2009 and 2017, seven children were diagnosed with HPS. A 0.4-year old infant with HPS-1 referred for the assessment of potential pulmonary involvement had no pulmonary symptoms and was excluded from this study. All the other cases were HPS-2.

The diagnosis of HPS-2 was based on typical clinical symptoms and proven by genetic analysis (Table 1). Mutational analysis was performed by Sanger sequencing. Routine clinical evaluation in different European centers was performed; data were collected retrospectively and prospectively following the inclusion into the study.

**Table 1:** Baseline demographics and genetics

	Patient 1	Patient 2	Patient 3	Patient 4	Patient 5	Patient 6	Distribution
Gender	female	female	male	female	female	female	5: 1, female: male
Consanguinity by history	yes	no	yes	no	yes, sibling of patient 1	no; genealogy demonstrated common ancestors, i.e. very distant relation	3: 3, yes: no
Alleles^a^	homozygous	homozygous	homozygous	compound eterozygous	homozygous	homozygous	5: 1, homozygous: compound heterozygous
*AP3B1* mutation 1	c.3222-3223delTG (^b^)	g.151312_159483del8172bp (^c^)	c.2546 T > G (^b^)	c.177delA (^b^)	c.3222-3223delTG (^b^)	c.2944delC (^b^)	
*AP3B1* mutation 2	c.3222-3223delTG (^b^)	g.151312_159483del8172bp (^c^)	c.2546 T > G (^b^)	c.1839_1842delTAGA (^b^)	c.3222-3223delTG (^b^)	c.2944delC (^b^)	
Previously described; predicted pathogenic effect of mutations	known [10]; likely pathogenic variant (frame shift)	known [28]; likely pathogenic variant (exon skipping)	unknown; likely pathogenic variant (stop-mutation; early termination in exon 22 instead of 27)	unknown; likely pathogenic variant (frameshift mutations and subsequent early terminations in exons 2 and 17)	known [10]; likely pathogenic variant (frame shift)	unknown, likely pathogenic variant (frame shift; early termination in exon 26)	2 known, 4 previously unknown variants

^a^All parents tested were heterozygous for the respective variants; not tested in patient 3. ^b^GenBank References (NM_003664.4) and ^c^(NG_007268.1)

Lung function testing was done according to standards set previously in children old enough to perform spirometry [13]. CT images of the chest were evaluated for the presence of parenchymal abnormalities (like mosaic attenuation, ground glass opacity, consolidation, linear opacity, septal thickening, reticular opacity, nodular opacity, honeycombing, emphysema, cysts, bleb or bulla) and airway abnormalities (tree-in-bud, bronchiectasis, bronchial wall thickening) on a lobar basis, counting lingula as the separate lobe [14]. Also the presence of pneumothorax, pleural thickening, pleural effusion and enlarged hilar or mediastinal lymph node were evaluated. The image analysis was performed blinded by a pediatric radiologist with expertise in chest imaging.

### Bronchoscopy and bronchoalveolar lavage (BAL)

Flexible Bronchoscopy including BAL (mostly of the middle lobe) were performed if clinically indicated using 3 times 1 ml warmed normal saline per kilogram body weight. BAL was examined cytologically and microbiologically.

### Lung biopsies and histological investigations

Lung biopsies available were peer-reviewed independently and blinded by a pathologist specialized in pulmonary pathology. Lung tissue of patient 3 was analyzed by Western blotting under reducing and denaturing conditions using sodium dodecyl sulfate–polyacrylamide gel electrophoresis followed by electroblotting and immunostaining for pro-SP-C (Merk Millipore, Darmstadt, Germany), ATF6, β-actin (abcam, Cambridge, UK), and cleaved caspase-3 (Cell Signaling, Gaithersburg, USA). Blotted membranes were developed with the ECL Plus chemiluminescent detection system (Amersham Biosciences, Amersham, UK). Immunohistochemistry was performed on lung tissue fixed in 4% formaldehyde on serial sections with the AP Fast Red kit (Zytochem Systems, Berlin, Germany) after antigen retrieval by microwaving in 10 mM sodium citrate buffer, pH 6.0. Hemalaun was used as counter-stain. Slides from patient 2 were also available for immunostaining for pro-SP-C and cleaved caspase-3, as described above. As controls, lung sections from 3 different organ donor lungs were used.

### Ethics, consent and permissions, consent to publish, declarations and statement

Informed consent to report individual patient data was obtained by all patients old enough to consent, and their parents or guardians. The study was approved by the ethics committee of the Ludwig-Maximilian University of Munich (EK 111-13).

All supporting data have been entered in the additional material (Additional file 1).

## Result

### Clinical course and mutations detected

The six patients included had a mean age at diagnosis of 8.83 years (Additional file 1: Detailed description of the individual cases. Subjects 1 to 6). On average, lung symptoms started 3.3 years before diagnosis (Table 2, Additional file 1: Table S1). Most frequent signs and symptoms reported at follow-up were dyspnea, coughing, need for oxygen, tachypnea, and clubbing. At last follow-up on average 3.17 years (range 2-6) after diagnosis, pulmonary symptoms were noted in all patients. Overall, the frequency of all pulmonary complaints increased over time. Non-pulmonary signs and symptoms are listed in Additional file 1: Table S1.

**Table 2:** Pulmonary signs and symptoms at diagnosis and last follow-up

	Patient 1	Patient 2	Patient 3	Patient 4	Patient 5	Patient 6	Mean (range) or distribution (range)/
Age [y] at start of lung symptoms	12^a^	2^b^	9^d^	0.8^e^	2	7^g^	5.5 (0.8-12)
Age [y] at clinical diagnosis/ last follow-up	12/ 17	9^c^/ 14	13/ 19	2/ 4	2/ 6	15/ 15	8.83 (2-15)/12 (4-19)
Dyspnea at diagnosis/ last follow-up	yes/ yes	no/ no	no/ during exercise	no/ recurrent	no/yes^1^	recurrent	1/ 5
Tachypnea at diagnosis/ last follow-up	no/ yes (32/min)	no/ no (16/min)	no/ no	no/ yes	no/ no	no/ no	0/ 2
Coughing at diagnosis/ last follow-up	yes/ yes	no/ no	no/ during exercise	no/ no	no/ yes	no/ no	1/ 3
Pneumothorax at diagnosis/ during course	no/ no	no/ yes (pleurodesis at age 12 y)	no/ yes (pleurodesis at age 15 y)	no/ no	no/ yes	no/ no	0/ 3
Clubbing at diagnosis/ last follow-up	no/ no	yes/ yes	yes/ yes	no/ no	nk/ nk	yes/ yes	3/ 3
Thoracic scoliosis at diagnosis/ last follow-up	no/ yes (since age 15 y)	yes/ yes (operative correction)	no/ yes (since age 13 y)	no/ no	yes/ yes	no/ no	2/ 4
Need for oxygen at diagnosis/ last follow-up	no/ no	no/ yes (night time)	no/no	no/ recurrent	yes/yes^f^	no/ no	1/ 3

Abbreviations: *y* year(s), *min* minute, *nk* no known, ^a^tachypnea without cough, sputum, or cyanosis, ^b^respiratory insufficiency and need of ventilation after birth, recurrent pneumothoraces during childhood, ^c^age at genetical diagnosis, ^d^at the age of 8 clubbing revealed, dyspnea and cough during exercise at the age of 9, ^e^recurrent pneumonia with need of oxygen, dyspnea and fever, ^f^with tracheostoma, ^g^several lower respiratory tract infections, clubbing revealed

**Fig. 1:**
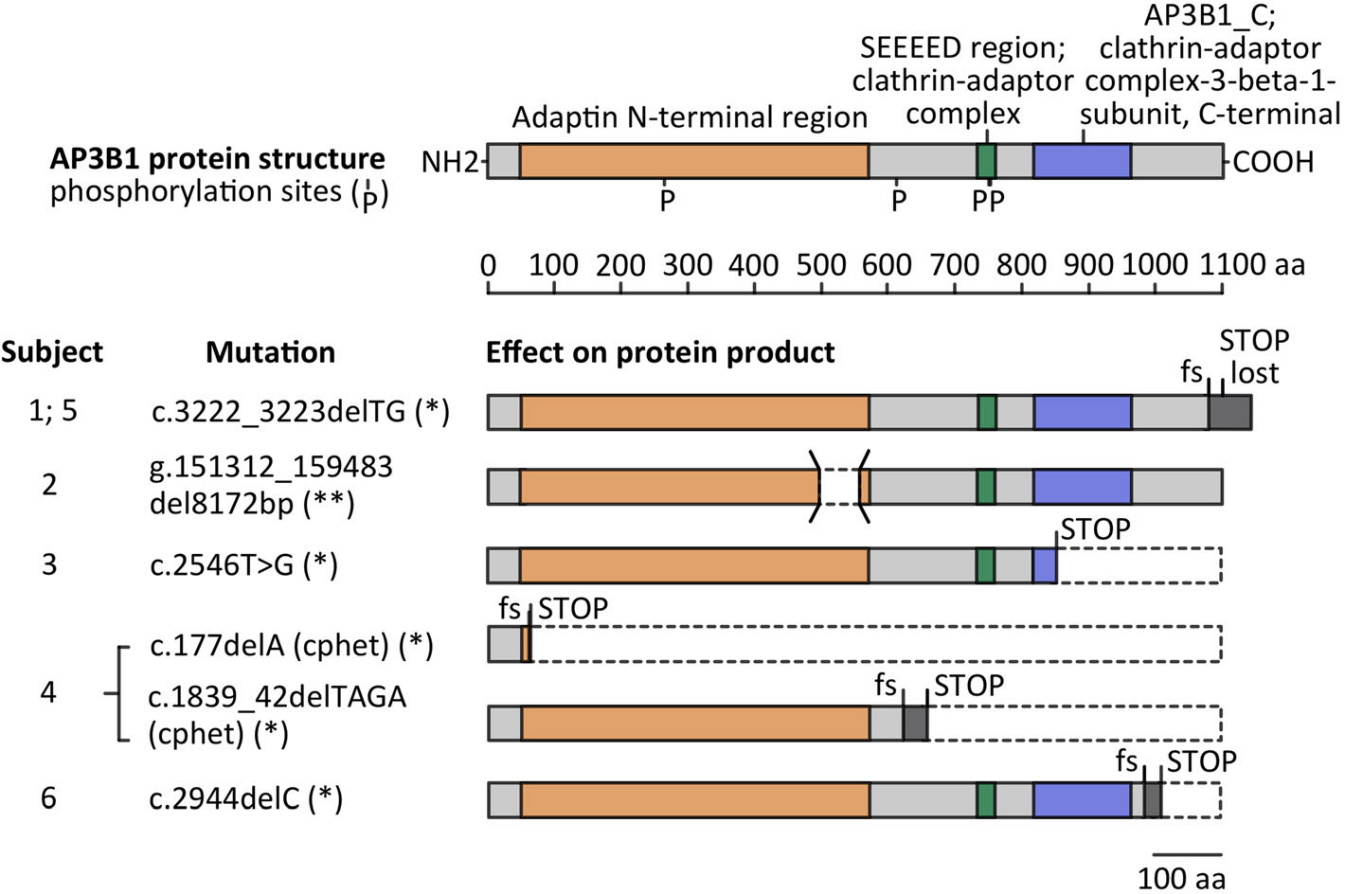
*AP3B1* mutations and their predicted effect on protein structure. Homozygous or compound heterozygous mutations found in individuals with HPS-2 are listed and the expected consequence for protein structure is illustrated. Colored segments represent relevant functional protein domains and regions of altered amino acid sequence after reading frame shift (grey). Genetic nomenclature refers to the respective entries in GenBank, NCBI. (*) NM_003664.4 (**) NG_007268.1 Abbreviations: aa = amino acid, cphet = compound heterozygous, fs = frame shift

Patients 1 and 5 were siblings and had the same homozygous frameshift mutation. All other children had different mutations in *AP3B1*, two of the variations were described before. All other mutations are likely disease-causing mutations, as they are predicted to result in truncated AP3B1 either through the introduction of a premature stop signal, by omission of a regular stop codon or by skipping of an important structural domain for adaptor protein 3 (AP-3 complex) formation. (Table 1, Fig. 1). Although a history of consanguinity could not be obtained in all cases, only one patient was compound heterozygous for the mutations, supporting the autosomal recessive pattern of inheritance (Table 1). Of interest, 5 of 6 patients were girls. Siblings with the same mutation had different clinical courses. However, the small size of this cohort precludes definite conclusions about genotype-phenotype associations in patients with HPS-2.

### Complications related to the respiratory tract

In 2 patients scoliosis was noticed at diagnosis, together with psychomotor retardation. Scoliosis developed during follow-up in 2 additional patients. In 3 patients, spontaneous pneumothorax occurred during follow-up. Pleurodesis was necessary for 2 patients (Table 2). In patients old enough to perform lung function testing, a slightly impaired diffusion capacity for carbon monoxide was noted. Initially, spirometry was in the mild abnormal range with combined restrictive- obstructive ventilation disorder, with changing course over time (Table 3, case descriptions in Additional file 1 and Additional file 2).

**Table 3:** Lung function measurements, lung biopsy results, treatments, and overall outcome

	Patient 1	Patient 2	Patient 3	Patient 4	Patient 5	Patient 6	Mean (n) or distribution
Age [y] at first / last lung function	12 / 17	nd (psychomotor retardation)	9 / 19	nd (too young)	nd (psychomotor retardation)	8/15	9.67 / 17 (3)
FEV1 [% predicted] first / last	74 / 71		82 / 66			63/73	73 / 70 (3)
FVC [% predicted] first / last	65 / 70		93 / 59			62/67	73.34 / 65.34 (3)
TLC [% predicted]	nd / 80		74 / 72				
DLCO [% predicted] (age at DLCO)	63 (15 y)/ 68 (17 y)		69 (15 y)/ 71 (19 y)				67.75 (4)
Lung biopsy	nd	cNSIP, UIP-like, DIP	UIP-like	nd	nd	cNSIP, DIP, lymphofollicular hyperplasia	3 biopsies
Chronic antibiotic treatment	Co-trimoxazole	Azithromycin	no	Azithromycin	Azithromycin	no	4/6
G-CSF s.c.	yes	yes	no	yes	yes	yes	5/6
Pirfenidone treatment	yes (started age 13 for 30 months)	no	yes (age 15 for 3 months)	no	no	no	2/6
Age last follow-up [y]	17	14	19	3.7	6	15	12.45 (range 3.7-19)
Overall outcome	sick-better	sick-same	sick-same	sick-same	died	sick-same	4 sick-same, 1 sick-better, 1 died

Abbreviations: *y* year(s), *FEV1* forced expiratory volume of first second, *FVC* forced vital capacity, *TLC* total lung capacity, *DLCO* diffusing capacity of the lung for carbon monoxide, *cNSIP* cellular non-specific interstitial pneumonitis, *nd* not done, *UIP-like* usual interstitial pneumonia- like features, *DIP* desquamative interstitial pneumonitis, *G-CSF* granulocyte-colony stimulating factor

### Chest imaging

**Fig. 2:**
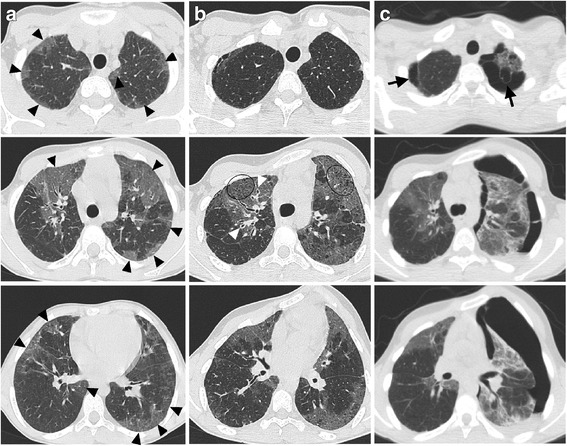
CT images of subject 3. **a**, **b**, **c** CT images at 8.7 years of age (column **a**) and follow-ups at age of 14.5 and 14.8 (column **b** and **c**). Leading pattern is GGO on both sides at initial scan (black arrowheads) and slight increase in reticular markings (encircled) and bronchial wall thickening (white arrowheads) at follow-up. Last follow-up showed distinctive pneumothorax and pleural effusion on the left and subpleural blebs (black arrows) in both lung apices

CT scans were performed at the time of diagnosis and at follow-up in subjects 1, 3 and 6 (Fig. 2, Additional file 3: Figure S1, Additional file 4: Figure S2, Additional file 5: Figure S4, Additional file 6: Figure S5, Additional file 7: Figure S6). For subject 4, CT was performed only at the time of diagnosis and for subject 2 only at follow-up. Age at first CT scan was 6.4 years (range 2.3-12) and 12.9 years (range 5.3-15.4) at follow-up. Leading radiographic pattern at diagnosis was ground-glass opacity, which had a patchy distribution, occurring in almost each lobe in all patients. In patient 1 also reticular opacities and traction bronchiectasis were detected at diagnosis. Patient 3 had bronchial wall thickening in the initial CT scan. There was a rapid increase in reticular pattern and traction bronchiectasis between initial and follow-up CT in all subjects with two examinations. Honeycombing and cysts were newly detectable in 3 patients after an observation time of 5.2 years. No nodular opacity, consolidation, tree-in-bud, emphysema, or air trapping were detected (Additional file 1: Table S2, Fig. 2, Additional file 3: Figure S1, Additional file 4: Figure S2, Additional file 5: Figure S4, Additional file 6: Figure S5, Additional file 7: Figure S6). There was no predominance of one side; upper lobes showed the most frequent affection.

### BAL and histology

Four of 6 patients had a BAL at diagnosis. One child had very mild eosinophilia and neutrophilia. Patient 6 had severe neutrophilia, despite peripheral blood neutropenia, suggesting compartmentalized capacity to mobilize neutrophils into the alveolar space (Additional file 1: Table S3). No pathogenic bacteria were recovered; patients were not on antibiotic treatment at the time of diagnosis.

**Fig. 3:**
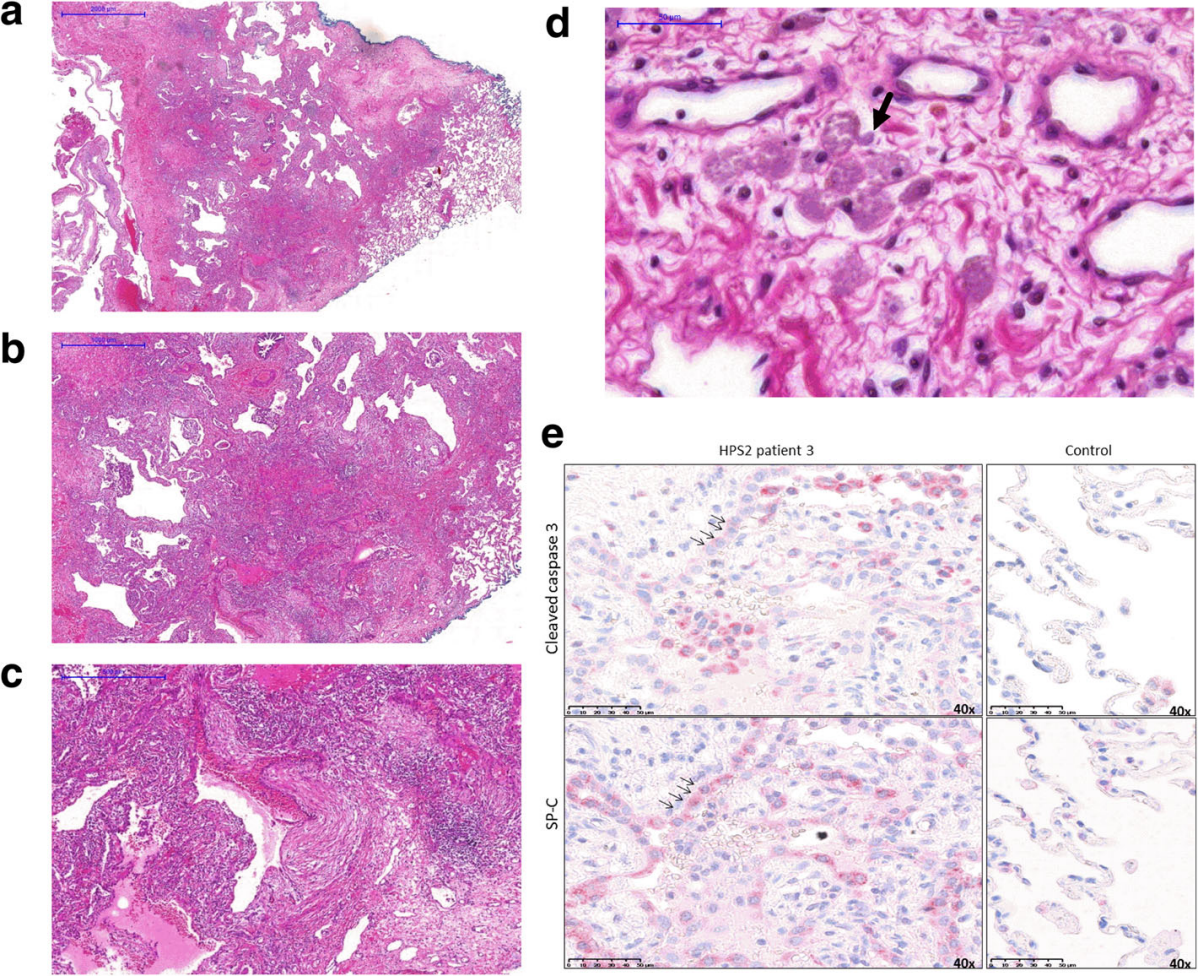
Pulmonary histopathology in subject 3. **a**-**d** Histological pattern of patient 3 shows patchy dense interstitial and pleural fibrosis with subpleural cysts/ blebs bordered by normal lung parenchyma at the right margin (**c**: HE × 10, **d**: fibrosis and cysts × 20) and small interstitial aggregates of ceroid macrophages with pale brown pigment in the cytoplasm (arrow) as a characteristic histological finding in HPS (D × 400). **e** Immunohistochemistry identifying alveolar type II cells by staining of proSP-C (SP-C), showing hyperplastic and vacuolated type II pneumocytes as another typical feature of HPS (see also in panel **d**). Increase in the apoptosis marker cleaved caspase-3 in alveolar epithelial type II cells. Representative images of immunohistochemistry for cleaved caspase-3 and proSP-C performed on serial paraffin sections of HPS-2 and organ donor lungs. Shown here are high magnification images (40×), indicating same type II cells stained for both cleaved caspase-3 and SP-C dying type II cells in HPS-2 patient lungs

Three of the 6 patients received a lung biopsy for diagnosis. Due to bleeding diathesis and low thrombocyte values, thoracoscopic biopsies were performed instead of transbronchial biopsies. In patient 2, the histological pattern of cellular non-specific interstitial pneumonitis (cNSIP), usual interstitial pneumonia- like features (UIP-like), and of desquamative interstitial pneumonitis (DIP) were noted and correlated to the pattern identified on chest CT (Additional file 4: Figure S2). Patient 3 had an UIP-like pattern of patchy dense fibrosis with subpleural cystic areas, pleural fibrosis, and pleural blebs. Histology showed hyperplastic and vacuolated type II pneumocytes and ceroid containing macrophages as typical features of HPS (see Fig. 3a-d). Patient 6 underwent lung biopsy at the age of 6 years. Comparable to patient 2 the histological pattern of cNSIP with areas of DIP were noted. Moreover, lymphofollicular hyperplasia with few lymphoid follicles was detectable. Typical vacuolated type II pneumocytes cells were found ubiquitously but only few ceroid containing macrophages (Additional file 8: Figure S7).

### Treatment

Because of neutropenia and resulting immune deficiency, chronic antibiotic treatment in 4 and Granulocyte-Colony Stimulating Factor, (G-CSF) treatment in 5 patients were initiated. The latter resulted in a lower normal range of neutrophil peripheral blood counts. Pirfenidone was used in 2 patients but was stopped after 3, respectively 24 months, due to gastrointestinal side effects and lack of sufficient clinical improvement (Table 3).

### Alveolar epithelial cellular stress and apoptosis

**Fig. 4:**
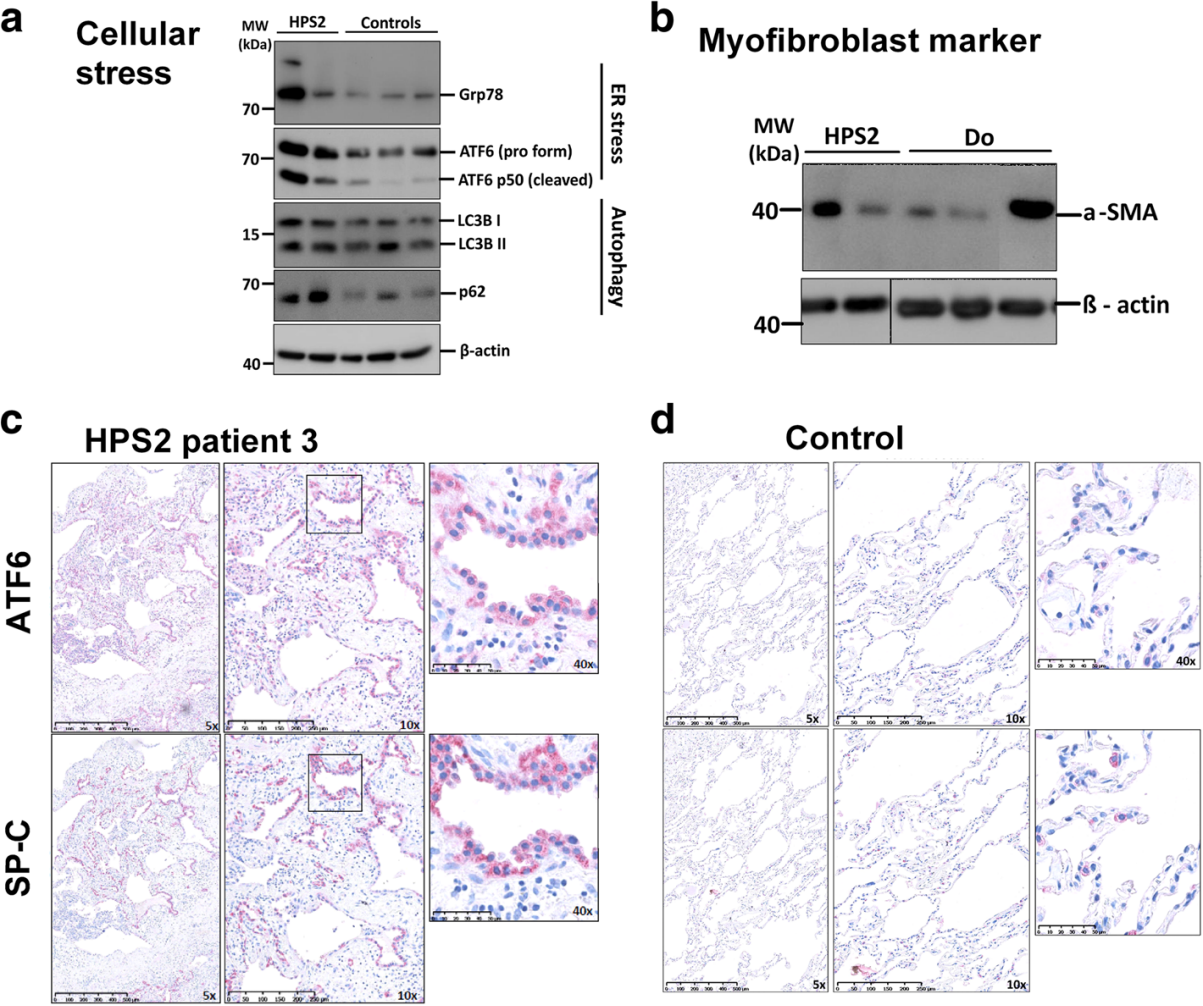
Activation of ER-stress and autophagy in subject 3. **a** Western blot images showing increased expression of the endoplasmic reticulum (ER) chaperone GRP78, ER stress marker AFT6, and importantly the p50 (cleaved form) of ATF6 in HPS-2 patient lungs. In addition, the autophagy marker LC3B (active lipidated form is LC3BII) and SQSTM1/p62 are concomitantly increased, indicating defective autophagy in HPS-2 patient lungs (HPS-2 = patient 3, samples from two different sites of a lung, controls = organ donors). **b** Representative Western blot images for the myofibroblast marker alpha-SMA and loading control, beta-actin in lung homogenates of patient 3 (HPS-2, duplicate lanes with samples from two different sites of a lung), and 3 different organ donors (controls, one lane each). **c** Increase in ER stress marker ATF6 in alveolar epithelial type II cells in HPS-2 patient lungs. Representative images of immunohistochemistry for ATF6 and proSP-C (SP-C, alveolar epithelial type II cells marker) performed on serial paraffin sections of HPS-2 and organ donor lungs. Shown here are low and high magnification images, using 5×, 10× and 40× objective for both HPS-2 and donor lung sections. Far right images indicate same alveolar epithelial type II cells stained for both ATF6 and SP-C indicating many alveolar epithelial type II cells positively stained for ATF6. Scale bar: as indicated in the images. **d** Control sections represent ATF6 and SP-C stainings in donor lung sections, where almost no ATF6 is detected in SP-C positive alveolar epithelial type II cells

In patient 3 with frozen tissue available obtained from 2 different parts of the lung, additional mechanistic investigations were performed and compared to healthy tissue obtained from unused lung of organ donors. The myofibroblast marker α- smooth muscle actin (SMA) was increased in one specimen supporting increased fibrosis (Fig. 4). Following whole lung tissue analysis, we further analyzed type II pneumocyte apoptosis, an important event in the pathogenesis of interstitial lung diseases. Serial sections revealed several type II pneumocytes to be positive for the apoptosis marker, cleaved caspase 3, indicating their apoptosis (Fig. 3e, Additional file 4: Figure S2H). Two types of cellular stress, the endoplasmic reticulum (ER) stress and autophagy can be differentiated in type II pneumocytes of HPS type 1 patient lungs [15, 16]. We thus analyzed the ER stress markers, GRP78 and p50 form of ATF6, which were markedly increased in one and moderately in the other specimen of the lungs of the HPS-2 patient (Fig. 4). Similarly, the autophagosomal marker microtubule-associated protein 1 light chain-3β, LC3B in its lipidated form (LC3BII) was elevated compared to two of the control lungs, one also gave an increased signal, along with a concomitant increase in the autophagy substrate protein, p62. This proved a defective autophagy pathway in the lung of this HPS-2 patient (Fig. 4a).

## Discussion

Children suffering from HPS-2 may present with a severe and progressive chronic pulmonary phenotype. Severe lung fibrosis may develop until early adulthood; associated complications including pneumothorax, recurrent infections, and the development of scoliosis were key features identified. Together with few forms of ATP-binding cassette sub-family A member 3 (ABCA3) [17–19], SFTPC [20, 21], and MARS deficiency [22], this condition operates under the few clearly and rapidly fibrosing diffuse parenchymal lung diseases in childhood.

Clinically HPS-2 in children is diagnosed by the combination of albinism, bleeding diathesis, and neutropenia. The evolution of symptoms in 4 children started with epistaxis or bleeding diathesis as initial symptoms during the first 2 years of life. However, respiratory symptoms were present in all our children but obviously were rated as too non-specific or developed too insidious to contribute to the diagnosis at age 5 years. At that time, half of our patients already had clubbing, dyspnea, and oxygen demand. Seventeen of the 22 cases under 18 years of age in the largest series on HPS-2 so far had respiratory symptoms, although not further specified [7]. Tachypnea and wet coughing developed somewhat later and suggest secondary chronic bronchitis or suppurative lung disease. The latter may be due to additional immune deficiency from neutropenia in HPS-2 subjects, which may aggravate early respiratory affections in these children. Three of our 6 patients developed pneumothoraces, adding substantially to pulmonary morbidity. Subpleural lung fibrosis, in particular cysts or honeycombing, predisposes to such a complication, which is very unusual in children at this young age. Among 4 children with HPS-2, Gochuico et al. described one child with 6 recurrent pneumothoraces [4].

The natural history of HPS-2 lung disease differs from that of HPS-1 pulmonary fibrosis, which usually affects middle-aged adults and not children [23]. Based on published reports, patients generally first manifest symptoms of HPS pulmonary fibrosis in middle age, however, anecdotal experience includes rare patients with HPS-1 beginning to develop ILD in late adolescence [24]. Carmona-Rivera described a 16-year-old boy with no pulmonary symptoms in HPS-1 [25]. Characteristic pulmonary CT findings known in adults with HPS-1 are increased reticular opacities, thickened interlobular septa, and ground-glass infiltrates in addition to fibrotic changes, including traction bronchiectasis, subpleural cysts and honeycombing. These imaging findings evolve over time, starting in early adulthood, as in HPS-1 patients < 20 years usually no CT changes are noted, in those between 20 and 29 years minimal changes were identified, with increasing symptoms in patients 30 years and older [26, 27]. In HPS-1, high-resolution CT abnormalities inversely correlated with percentage of forced vital capacity and were useful in defining the progression of interstitial disease [27]. In our patients with HPS-2, patchy ground-glass opacity predominated at diagnosis during early childhood and a reticular pattern evolved rapidly over time. At follow-up most of the children developed the CT findings characteristic for HPS fibrosis in adults. Thus, compared to patients with HPS-1, in HPS-2 not only pulmonary symptoms as described above but also CT abnormalities were detected very early. Of interest and in contrast to our observations, two children with HPS-2 have been described in literature before with bilateral ground-glass opacity, thickening of interlobular septa, and interstitial reticulations (4 and 8 vs. 14 years of age) and 3 more children suffering from HPS-2 had changes in HRCT all with improvement over time [4, 9].

In all children, the diagnosis of HPS-2 was verified genetically (Table 1). A pair of siblings had the same mutation (patient 1 and 5), however their clinical course was different. This was also due to the fact that the pulmonary phenotype of the second child was much more and earlier in focus after the other died. A frameshift mutation in the C-terminal region of *AP3B1* resulted in loss of the stop codon, prolonging translation into the 3’UTR region. Although an alternative in-frame stop codon is available further downstream, increased distance to the original stop codon may predispose transcripts to nonstop-mediated decay mechanisms [28]. Alternatively, the translated protein product might be subject to proteolytic breakdown due to misfolding, defective assembly, or intrinsic conformational instability [29]. Patient 2 displayed a larger genomic deletion which includes parts of introns 14, 15, and exon 15. This specific region has shown to be essential for correct assembly of the AP3-complex [30]. In all other individuals, single point mutations (patient 3) or frameshift mutations caused by base pair deletions about 10-120 base pairs upstream (patient 4, 6) lead to premature stop codons, translation termination, and possibly activation of the nonsense-mediated decay pathway (Fig. 1).

The pulmonary fibrosis in patients with HPS may be preceded by a macrophage-mediated alveolar inflammation, as BAL fluid contains increased numbers of constitutively activated macrophages [26]. Only one of the subjects with BAL had increased macrophage counts (Additional file 1: Table S3). All our patients had progressively fibrosing lung disease already during childhood. Our histological data prove that pulmonary fibrosis affects children with HPS-2. This is in contrast to HPS-1 and 4, where the development of pulmonary fibrosis starts in middle-age adults and children are only rarely affected [24]. Overall rapidly progressive fibrosing lung disease in childhood is extremely rare. The development of fibrosis might be facilitated by the patients’ neutropenia and natural killer - and T-cell dysfunction and the resulting susceptibility to severe recurrent chest infections. Such an observation is consistent with the HPS-2 animal model where environmental lung injury by silica or bleomycin aggravates fibrosis [26]. Therefore, preventive measures as vaccination and aggressive antibiotic treatment are warranted. Taking our limited observation length into account, the clinical course was not stable in most children. Despite intense symptomatic treatments after diagnosis, we saw deterioration and development of complications in several patients. Four patients remained unchanged, one patient improved, one patient (subject 5), however, died from respiratory insufficiency.

The histology of HPS-2 interstitial lung disease is not widely explored, as the diagnosis may now be done genetically. Patient 2 had a combination of NSIP and UIP-like pattern with dense fibrosis in peribronchiolar and subpleural distribution, as well as a DIP- like areas with intraalveolar aggregates of alveolar macrophages, very similar to other descriptions published [4]. Lung biopsy of patient 3 was dominated by a patchy dense fibrosis with UIP-like pattern comparable to patient 2. Lung biopsy of patient 6 did not show areas of dense fibrosis but demonstrated a cellular NSIP pattern. Additionally, there was a mild lymphoid hyperplasia with few lymphoid follicles with germinal centers that could probably be interpreted as post-infectious changes.

Apoptosis of type II pneumocytes, in addition to ER stress and defective autophagy, was observed in a HPS-2 patient lung. This finding is in line with the previously reported observations of cellular stress and apoptosis of type II pneumocytes in several interstitial lung diseases. More studies are needed to determine if defective autophagy or ER stress underlie type II pneumocyte apoptosis and are subsequently responsible for fibrotic remodeling in the HPS-2 patient lung.

## Conclusion

We highlight that patients with HPS-2 in contrast to the other forms of HPS must be considered a severely and rapidly fibrosing lung disease already during early childhood which requires effective antifibrotic treatment. The latter is not yet available in childhood. At diagnosis, half of the subjects had clinical signs of chronic hypoxemia. Histology remains of importance in those extreme rare diseases, which should be made based on the clinical phenotype and confirmed by targeted genetics. During the course, which may be complicated by recurrent pneumothoraces and scoliosis, a comprehensive multidisciplinary team approach is needed. Currently early symptomatic pulmonary care including consequent antibiotic treatment of pulmonary infections and vaccinations. Nutritional management and early orthopedic treatment of scoliosis may help to improve the outcome of children with HPS-2. No specific antifibrotic treatment is available in children.

## Additional files

Additional file 1: Detailed description of the individual cases. Subjects 1 to 6. **Table S1.** Non-pulmonary signs and symptoms. **Table S2.** Blinded scoring of CT-scans (number of CTs available at diagnosis (n = 5)/ at follow-up (n = 5); CTs at follow-up and diagnosis available in sub 1-3 and 6, only at follow-up in sub. 2 and only at diagnosis in sub. 4). Scans were analysed for the presence and absence of features listed in the six sections of the lungs. Scoring was not validated. **Table S3.** Results of bronchoalveolar lavage (BAL) at diagnosis. (DOC 107 kb)

Additional file 2: **Figure S3.** Bodyplethysmography of subject 3. Bodyplethysmography at the last follow up (19 years of age). Visible is the restrictive pattern with mild obstruction. (TIFF 807 kb)

Additional file 3: **Figure S1.** CT scan and spirometry course of subject 1. (**A**) CT scan at 12 and 15 years of age. Initially patchy GGO (black arrowheads) and mild reticulations were seen in all lobes with apical predominance, at follow-up also reticular pattern increased and traction bronchioloectasis and mild honeycombing appeared (black arrows). (**B**) Spirometry long-term course of FVC which shows variabilities of 15% during the last 4 years. Pirfenidone treatment led to improvement first, but had no effect during long term course. First bodyplethysmographia was performed at 13 years of age, last one at the age of 17 years. (TIFF 3981 kb)

Additional file 4: **Figure S2.** CT scan and histological pattern of subject 2. (**A**) CT scan at the age of 13 8/12 years. CT images show the thorax deformity due to scoliosis with displacement of the central bronchial structures and traction of the segmental bronchi. On the left side, cystic parenchymal destruction in the apex (black arrow), mild bronchial wall thickening in the upper lobe (encircled), reticular opacities predominantly in the upper lobe and lingula. Increased lung density especially in the lower lobe is due to breathing artefacts and areas of ground-glass opacity. On the right side, there are few subpleural cysts in the apex and reticular opacities in the upper and middle lobe. (**B**-**G**) Histological pattern with patchy areas of dense interstitial fibrosis (red) in peribronchiolar and subpleural distribution (B: HE, x10). Dense fibrosis with small areas of honeycombing with bronchiolar metaplasia (C: HE, x50). Fibroblastic foci as features of UIP pattern (D: HE, x 200) and areas with NSIP pattern consisting of diffuse mild interstitial fibrosis and a mild chronic inflammatory infiltrate in between (E: HE, x100). Few small interstitial aggregates of ceroid macrophages (F: HE, x200) and vacuolization of hyperplastic type II pneumocytes (G: HE, x400) as subtle but characteristic features of HPS (G). (H) Increase in the apoptosis marker cleaved caspase-3 in alveolar epithelial cells type II (AECII) in HPS2 patient lungs. Representative images of immunohistochemistry for cleaved caspase-3 and proSP-C (AECII marker) performed on serial paraffin sections of HPS2 and organ donor lungs. Shown here are high magnification images, 40x objective for both HPS2 and donor lung sections. Arrows indicate same AECII stained for both cleaved caspase-3 and SP-C implying dying AECII in HPS2 patient lungs while almost no cleaved caspase-3 is detected in SP-C positive AECII of donor lungs. Scale bar: as indicated in the images. (TIFF 4239 kb)

Additional file 5: **Figure S4.** CT scan of subject 4. CT Scan with patchy distribution of ground glass opacity throughout all lobes. (JPEG 8908 kb)

Additional file 6: **Figure S5.** CT scan of subject 5. CT at 2 (left column) and 5 (right column) years of age. Initial scan was performed in prone position, follow up in supine position. Initial scan demonstrates linear opacities (black arrows) with mild traction bronchiectasis. After a course of 3 years CT scan deteriorates with severe parenchymal abnormalities showing diffuse ground glass opacity, reticular opacity, mild honeycombing, and cysts (black arrowheads). Moreover enlarged mediastinal und hilar lymph nodes have been seen (better seen on the mediastinal window, images not provided here). (TIFF 7675 kb)

Additional file 7: **Figure S6.** CT scan of subject 6. CT images at 6 (first column), 9 (second column), 11 (third column) and 15 (fourth column) years of age. At the initial scan subject presented with patchy GGO in all lobes and additional interlobular septal thickening (encircled in white). At first follow up, a decrease of GGO in the left upper and lower lobes can be seen (black arrows in initial scan, first column). Over the next years, the distribution and extent of GGO showed no relevant changes. The extents of interlobular septal thickening decreased at the first follow up, followed by increase of reticular markings (encircled in black) in the next scans. Also bronchial wall thickening was present on the last follow up scans, here best seen in the right lower lobe in the third column (dashed circle). (TIFF 4655 kb)

Additional file 8: **Figure S7.** Histological pattern of subject 6. A (HE, x50): Normal lung architecture with partly collapsed alveolar spaces on the right containing aggregates of alveolar macrophages (Black arrows). Hypercellular interstitium with diffuse mild lymphocytic infiltrate in alveolar septa and few lymphoid follicles. B (HE, x100): Prominent lymphoid follicle with central germinal center, interstitial lymphocytes and intraalveolar macrophages. C (HE, x200): Aggregates of intraalveolar macrophages with pink cytoplasm (black arrows), interstitial lymphocytes with small blue nuclei (black triangles), scattered type II pneumocytes with clear vacuolated cytoplasm (white arrows) typically found in HPS. (TIFF 5371 kb)

## Abbreviations

ABCA3: ATP-binding cassette sub-family A member 3
AP3: Adaptor protein 3
*AP3B1*: Adapter protein 3 complex subunit beta-1
ATF6: Activating transcription factor 6
BAL: Bronchoalveolar lavage
cNSIP: Cellular non-specific interstitial pneumonitis
CT: Computer tomography
DIP: Desquamative interstitial pneumonitis
ER: Endoplasmic reticulum
G-CSF: Granulocyte-Colony Stimulating Factor
GRP78: Glucose-regulated protein 78
HPS: Hermansky-Pudlak syndrome
ILD: Interstitial lung disease
LC3BII: Light chain-3β, LC3B in its lipidated form
MARS: Methionyl-tRNA Synthetase
mM: Millimolar
p50: Protein 50
p62: Protein 62
SMA: Smooth muscle actin
SP: Surfactant protein
UIP: Usual interstitial pneumonia
