# Supplementary figures and images for: Hermansky-Pudlak syndrome type 2 manifests with fibrosing lung disease early in childhood
Source: Orphanet J Rare Dis. 2018 Mar 27;13:42. doi: 10.1186/s13023-018-0780-z (PMC5870397; doi:10.1186/s13023-018-0780-z)

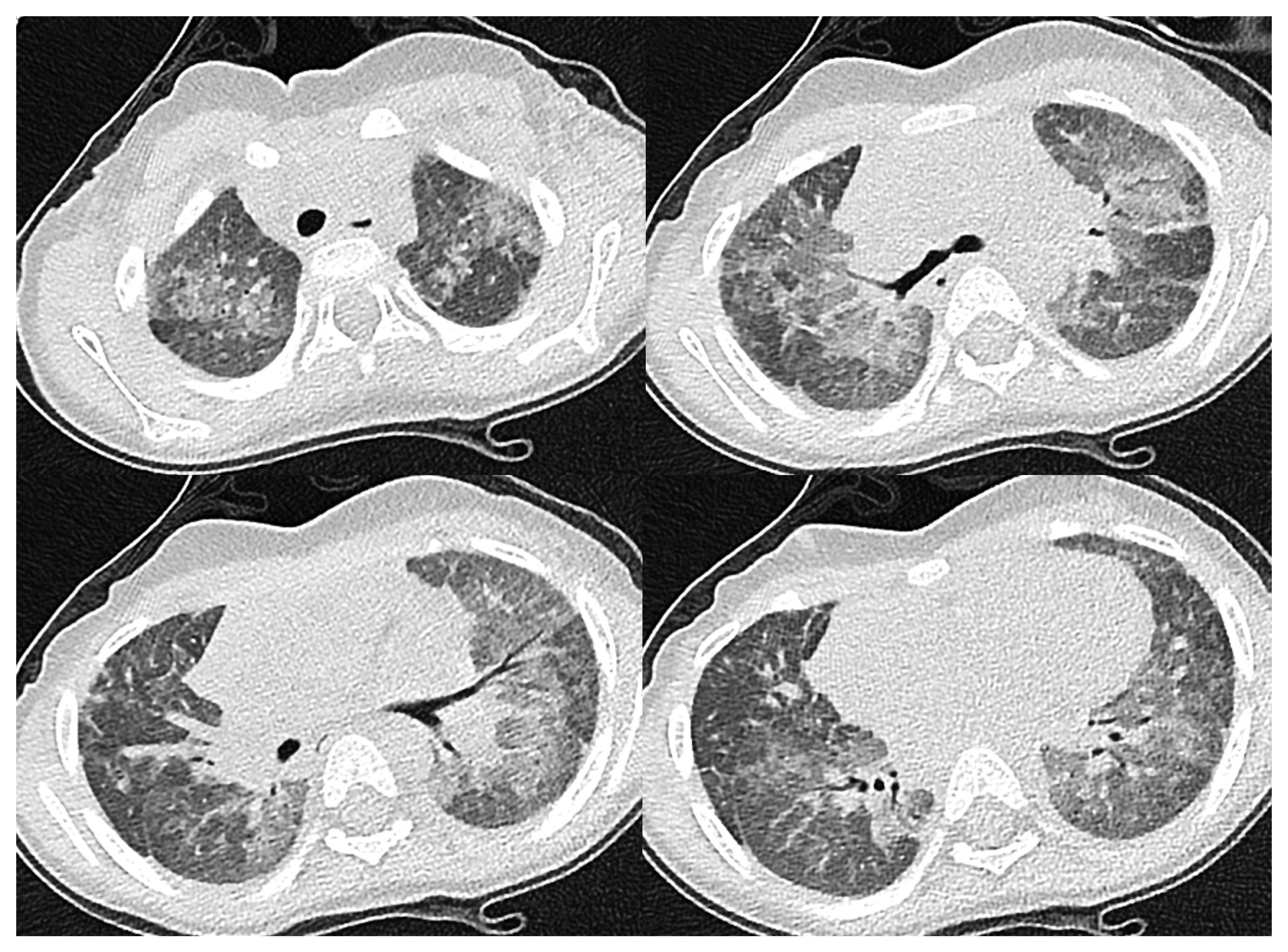

Supplement: Supplementary file 5 — Figure S4. CT scan of subject 4. CT Scan with patchy distribution of ground glass opacity throughout all lobes. (JPEG 8908 kb) [file 13023_2018_780_MOESM5_ESM.jpg]
