# Supplementary material for: Hermansky-Pudlak syndrome type 2 manifests with fibrosing lung disease early in childhood
Source: Orphanet J Rare Dis. 2018 Mar 27;13:42. doi: 10.1186/s13023-018-0780-z (PMC5870397; doi:10.1186/s13023-018-0780-z)
Supplement: Supplementary file 1 — Detailed description of the individual cases. Subjects 1 to 6. Table S1. Non-pulmonary signs and symptoms. Table S2. Blinded scoring of CT-scans (number of CTs available at diagnosis (n = 5)/ at follow-up (n = 5); CTs at follow-up and diagnosis available in sub 1-3 and 6, only at follow-up in sub. 2 and only at diagnosis in sub. 4). Scans were analysed for the presence and absence of features listed in the six sections of the lungs. Scoring was not validated. Table S3. Results of bronchoalveolar lavage (BAL) at diagnosis. (DOC 107 kb) [file 13023_2018_780_MOESM1_ESM.zip › HPS additional file 1 table 1.doc]

**Additional Table 1**: Non-pulmonary signs and symptoms

|  | subject 1 | subject 2 | subject 3 | subject 4 | subject 5 | subject 6 |
| --- | --- | --- | --- | --- | --- | --- |
| age at onset of extrapulmonary symptoms / pulmonary symptoms [y] | 8/ 12 | 2/ 2 | 3/ 9 | 1/ 1 | 1.4/ 2 | 5/ 7 |
| clinical diagnosis - pulmonary symptoms [y] | 0^a^ | 7^#^ | 4 | 1 | 0 | 6 |
| albinism skin / ocular | yes / no | yes / no | partial / yes | yes / yes | yes / no | yes /yes |
| ophthalmological pathology | myopia | no | no | no | no | myopia |
| hearing loss | no | yes | yes | no | nk | no |
| diarrhea | no | no | no | no | no | no |
| failure to thrive | yes | yes (PEG) | no | yes (PEG) | yes (PEG) | no |
| bleeding history | yes | yes | no | no | yes | yes |
| low platelets count [< 150 G/l]  platelet count at last follow up | no  180 | no  154 | yes  106 | yes  289  minimal 135 | no  175  minimal 131 | yes  nk |
| neutropenia [< 500/µl]  neutrophil count absolute [normal range: 1.82-7.47/µl]  neutrophil count relative [normal range: 40-75%]  minimal neutrophil count | yes   0,82*  36%*  0,19/µl | yes  nk  79%*  5% | yes  nk | yes  1,27*  31%*  1,25/µl | yes  nk | yes  nk |

Abbreviations: PEG = percutaneous gastrostomy, y = year(s), ^a^diagnosis due to familial genetic screening, ^#^age at genetic diagnosis, G/l= Giga per liter, µl= microliter, *at last follow up
