# Supplementary material for: Hermansky-Pudlak syndrome type 2 manifests with fibrosing lung disease early in childhood
Source: Orphanet J Rare Dis. 2018 Mar 27;13:42. doi: 10.1186/s13023-018-0780-z (PMC5870397; doi:10.1186/s13023-018-0780-z)
Supplement: Supplementary file 1 — Detailed description of the individual cases. Subjects 1 to 6. Table S1. Non-pulmonary signs and symptoms. Table S2. Blinded scoring of CT-scans (number of CTs available at diagnosis (n = 5)/ at follow-up (n = 5); CTs at follow-up and diagnosis available in sub 1-3 and 6, only at follow-up in sub. 2 and only at diagnosis in sub. 4). Scans were analysed for the presence and absence of features listed in the six sections of the lungs. Scoring was not validated. Table S3. Results of bronchoalveolar lavage (BAL) at diagnosis. (DOC 107 kb) [file 13023_2018_780_MOESM1_ESM.zip › HPS additional file 1 table 2.doc]

**Additional Table 2**: Blinded scoring of CT-scans (number of CTs available at diagnosis (n=5)/ at follow-up (n=5); CTs at follow-up and diagnosis available in sub 1-3 and 6, only at follow-up in sub. 2 and only at diagnosis in sub. 4). Scans were analysed for the presence and absence of features listed in the six sections of the lungs. Scoring was not validated.

|  |  | right lung | RUL  (initial/ follow-up) | ML  (initial/ follow-up) | RLL  (initial/ follow-up) | left lung | LUL  (initial/ follow-up) | Lingula  (initial/ follow-up) | LLL  (initial/ follow-up) |
| --- | --- | --- | --- | --- | --- | --- | --- | --- | --- |
| pleura | pleural effusion |  |  |  |  | 1/0 |  |  |  |
|  | pneumothorax |  |  |  |  | 0/1 |  |  |  |
| mediastinum | enlarged mediastinal lymph nodes | 1/1 |  |  |  | 0/1 |  |  |  |
|  | enlarged hilar lymph nodes | 0/1 |  |  |  | 0/1 |  |  |  |
| parenchyma | GGO diffuse |  | 0/1 | 0/1 | 0/1 |  | 0/1 | 0/1 | 0/1 |
|  | GGO patchy |  | 4/3 | 4/4 | 4/3 |  | 4/4 | 4/3 | 4/4 |
|  | linear opacity |  | 0/1 |  |  |  |  |  |  |
|  | reticular opacity |  | 1/4 | 0/3 | 0/2 |  | 1/4 | 1/4 | 0/4 |
|  | honeycombing |  | 0/2 | 0/1 | 0/1 |  | 0/3 | 0/1 | 0/1 |
|  | cyst/ subpleural bulla |  | 0/4 | 0/2 |  |  | 0/3 | 0/2 |  |
|  | septal thickening |  | 1/0 | 1/0 | 1/0 |  | 1/0 | 1/0 | 1/0 |
| airways | traction bronchiectasis/ architectural distortion |  | 1/4 | 0/2 | 0/1 |  | 1/3 | 1/2 | 0/1 |
|  | bronchial wall thickening |  | 1/2 | 0/1 | 0/1 |  | 0/1 |  |  |

Abbreviations: GGO: ground-glass opacity; RUL: right upper lobe, ML: middle lobe, RLL: right lower lobe, LUL: left upper lobe, LLL: left lower lobe.
