# Supplementary material for: Hermansky-Pudlak syndrome type 2 manifests with fibrosing lung disease early in childhood
Source: Orphanet J Rare Dis. 2018 Mar 27;13:42. doi: 10.1186/s13023-018-0780-z (PMC5870397; doi:10.1186/s13023-018-0780-z)
Supplement: Supplementary file 1 — Detailed description of the individual cases. Subjects 1 to 6. Table S1. Non-pulmonary signs and symptoms. Table S2. Blinded scoring of CT-scans (number of CTs available at diagnosis (n = 5)/ at follow-up (n = 5); CTs at follow-up and diagnosis available in sub 1-3 and 6, only at follow-up in sub. 2 and only at diagnosis in sub. 4). Scans were analysed for the presence and absence of features listed in the six sections of the lungs. Scoring was not validated. Table S3. Results of bronchoalveolar lavage (BAL) at diagnosis. (DOC 107 kb) [file 13023_2018_780_MOESM1_ESM.zip › HPS additional file 1 text.doc]

**Additional material**

**Detailed description of the individual cases**

**Subject 1** was diagnosed because of a familial screening. Her sister (subject 5) died at the age of 6 years. The consanguine, healthy parents have 3 other healthy girls and are of Arabic origin. She was under frequent clinical examination due to recurrent bleeding diathesis with slightly decreased platelet counts. Due to recurrent epistaxis, cauterization was performed at the age of 8 years. She had albinism and recurrent infections due to neutropenia so that a co-trimoxazole prophylaxis was performed twice weekly. Moreover, she had granulocyte colony-stimulating factor (G-CSF) treatment during infections. She needs glasses due to myopia.

She suffers from tachypnea without cough, sputum, or cyanosis, but she had no recurrent lower respiratory tract infections. At the age of 12 years, lung fibrosis was first suspected as a HRCT was performed due to a respiratory exacerbation. Moreover, ground-glass opacity (GGO) and reticular pattern (Additional Fig. 1A) were found.

At her last follow up at the age of 17 years, lung function revealed a restrictive pattern: Total Lung Capacity (TLC) 3.03 l, 80% predicted; Forced Vital Capacity (FVC) 2.25 l, -2.43 z-scores; Forced Expiratory Pressure in 1 Second (FEV1) 2.04 l, -2.48 z-scores) and Diffusing Capacity of the lung for Carbon Monoxide (DLCO) was decreased to 68% (Additional Fig. 1B). The clinical status and radiological findings were stable during the last years.

**Subject 2** is a now 15-year-old girl. She had respiratory insufficiency and need of ventilation after birth in combination with albinism. During childhood, she developed bullous lung disease with recurrent pneumothoraces so that pleurodesis was performed in 2013. A lung biopsy performed earlier showed increased macrophages, vacuolated type 2 pneumocytes, atypical lamellar bodies, bullous and atelectatic lung parenchyma, but only small, densely packed patches of subpleural fibrosis besides areas of mild diffuse interstitial fibrosis and mild chronic interstitial inflammation. The findings were described as NSIP, UIP-like, and DIP-like patterns (Additional Fig. 2 B-G). High-resolution CT during follow up at the age of 13 showed bullous changes and honeycombing, but no GGO or bronchiectasis (Additional Fig. 2 A). Beside that, she had no pulmonary symptoms and no desaturations during sleep or exercise.

Due to neutropenia, she was treated with Zithromax and G-CSF three times weekly..

Since 2011, she is feed via Percutaneous Endoscopic Gastrostomy (PEG) tube due to dystrophy. In addition, she has hearing loss, psychomotor retardation of unknown origin, and scoliosis with the need for several operative treatments. Due to the retardation, no lung function test has been performed. At her last follow up at the age of 15 years, she is sick-same with rare pulmonary exacerbation and the need for oxygen during exercise and sleep.

**Subject 3** is the only male in our cohort. He is the second child of healthy parents. Consanguinity was positive. At the age of 2 years, he was admitted due to Purpura Schönlein-Henoch and neutropenia was revealed without recurrent infections or clinical signs of immunodeficiency. At the age of 8 years, clubbing led to the diagnosis of an interstitial lung disease. A CT scan was performed and revealed GGO. A second CT-scan in 2011 showed a progression with traction bronchiectasis (Fig. 2A,B). At that time, lung function testing was performed and revealed a restrictive pattern with TLC 72 and VC of 59% predicted (Additional Fig. 3). DLCO was reduced to 69%. At that age, he developed pulmonary symptoms, like dyspnea and cough during exercise. One year later, a hearing loss was detected and HPS was suspected, as an ocular albinism existed. Only 4 months after the second CT scan, recurrent pneumothoraces occured and pleurodesis was performed in combination with lung biopsy (Fig. 2C). Lung biopsy showed an UIP-like pattern of patchy dense interstitial fibrosis surrounded by normal lung parenchyma associated with subpleural cystic airspaces and pleural blebs (Fig. 3A-D). He had no recurrent bleeding diathesis but platelet function test showed platelet storage pool defect. Moreover, he has scoliosis but without need for any operative intervention.

At the end of 2012, pirfenidone treatment was started but ended at an early stage after 3 months of treatment, as no subjective or objective success was detectable, but abnormal fatigue appeared.

**Subject 4** is a now 4.1-year-old female with oculo-cutaneous albinism, congenital dislocation of the hips, necrotizing fasciitis, neutropenia, and a thrombocyte storage pool disease without severe bleeding problems until now. Immunologic testing showed normal T- and B-cell subsets and low NK-cells. She receives G-CSF with now normal neutrophil counts. She has been admitted repeatedly with dyspnea, oxygen need, and fever. Pneumonia was diagnosed 4 times until now. Between hospital admissions, she has tachypnea with normal oxygen saturation. Chest X-rays showed bilateral consolidative abnormalities that did not improve over time despite antibiotic courses and antibiotic maintenance therapy. Chest CT was performed at the age of 2 years which showed extensive bilateral ground-glass opacities (Additional Fig. 4). Normal amounts and molecular forms for SP-B and SP-C have been found with no evidence for hydrophobic surfactant system deficiency.

**Subject 5**, the younger sister of subject 1, had no noticeable problems until the age of 16 months. At that time, a tonsillectomy was performed and a severe bleeding with need for a cardiopulmonary reanimation took place at the 11. postoperative day. In consequence, she unfortunately developed a severe global cerebral damage with hypoxic cerebral paresis and epilepsy. Additionally, a PEG needed to be installed, as normal feeding was impossible. Later on, she developed a severe gastroesophageal reflux, so that a fundoplication was done.

At the age of 2 years, HPS-2 was diagnosed in the Arab Emirates due to recurrent epistaxis in combination with an albinism.

At the age of 4 years, she had a permanent need for oxygen and ventilation after pneumonia so that tracheostomy was performed. A lung CT scan performed at the age of 5 showed severe parenchymal destruction with diffuse ground-glass opacity, reticular opacity, honeycombing and cysts (Additional Fig. 5). After tracheostomy, she developed an acute respiratory distress syndrome (ARDS) and continuous respiratory aggravation due to either infections or hypoventilation. During that phase, she developed a pneumothorax once. Treatments with invasive ventilation, recurrent relaxation and sedation, as well as antibiotic treatments were without success. 2 steroid pulses led to temporary improvement. Unfortunately, she died at the age of 6 years due to respiratory insufficiency.

**Subject 6** is a now 15-year-old female with oculocutaneous albinism, neutropenia, and a thrombocyte storage pool defect. At birth, she had respiratory difficulties and was treated in a neonatal intensive care unit with mechanical ventilation for 10 days. She was closely monitored from birth because of neutropenia. She has had episodes with epistaxis and is easily bruised. Immunologic testing has shown normal B- and T-cell populations and low NK-cells. She has been treated with G-CSF, which resulted in normal neutrophil count, but does not receive this treatment continuously. A CT at 6 years of age showed extensive GGO and interlobular septal thickening with increase of reticular marking at follow-ups (Additional Fig. 6). Despite extensive investigations, a definite diagnosis was first made at the age of 15 years. She has some dyspnea during activity, but her measured lung function has been stable. Tachypnea has only been observed around the age of 6-7 when she had several lower respiratory tract infections. Moreover, clubbing was noticed at 7 years of age and has remained unchanged. During follow-up she had recurrent respiratory tract infections (otitis, tonsillitis, bronchitis, and pneumonia) and one case of orbital cellulitis, but fewer in the last years. Lung biopsy results are shown in Additional Fig. 7.
